# A time series analysis of weather variability and all-cause mortality in the Kasena-Nankana Districts of Northern Ghana 1995–2010

**DOI:** 10.3402/gha.v5i0.19073

**Published:** 2012-11-23

**Authors:** Daniel K. Azongo, Timothy Awine, George Wak, Fred N. Binka, Abraham Rexford Oduro

**Affiliations:** 1 Navrongo Health Research Centre, Ghana Health Service, Navrongo, Ghana; 2 INDEPTH-Networks, Accra, Ghana; 3 University of Health and Allied Sciences, HO, Ghana

**Keywords:** mortality, temperature, precipitation, time series analysis, distributed lag model, season

## Abstract

**Introduction:**

Climate and weather variability can have significant health consequences of increased morbidity and mortality. However, today the impact of climate and weather variability, and consequentially, of climate change on population health in sub-Saharan Africa is not well understood. In this study, we assessed the association of daily temperature and precipitation with daily mortality by age and sex groups in Northern Ghana.

**Methods:**

We analysed daily mortality and weather data from 1995 to 2010. We adopted a time-series Poisson regression approach to examine the short-term association of daily mean temperature and daily mean precipitation with daily mortality. We included time factors and daily lagged weather predictors. The correlation between lagged weather predictors was also considered.

**Results:**

For all populations, a statistically significant association of mean daily temperature with mortality at lag days 0–1 was observed below and above the 25th (27.48°C) and 75th (30.68°C) percentiles (0.19%; 95% confidence interval CI: 0.05%, 0.21%) and (1.14%; 95% CI: 0.12%, 1.54%), respectively. We also observed a statistically significant association of mean daily temperature above 75th percentile at lag days 2–6 and lag days 7–13 (0.32%; 95% CI: 0.16%, 0.25%) and (0.31% 95% CI: 0.14%, 0.26%), respectively. A 10 mm increase in precipitation was significantly associated with a 1.71% (95% CI: 0.10%, 3.34.9%) increase in mortality for all ages and sex groups at lag days 2–6. Similar results were also observed at lag days 2–6 and 14–27 for males, 2.92% (95% CI: 0.80%, 5.09%) and 2.35% (95% CI: 0.28%, 4.45%).

**Conclusion:**

Short-term weather variability is strongly associated with mortality in Northern Ghana. The associations appear to differ among different age and sex groups. The elderly and young children were found to be more susceptible to short-term temperature-related mortality. The association of precipitation with mortality is more pronounced at the short-term for all age and sex groups and in the medium short-term among males. Reducing exposure to extreme temperature, particularly among the elderly and young children, should reduce the number of daily deaths attributable to weather-related mortality.

Climate and weather variability can have significant health consequences, resulting in increases in morbidity and mortality (1–3). However, their impact on people living in tropical regions has not been as well described. The prevention of deaths caused by extreme weather conditions, including temperatures and precipitation is an issue of importance concerning public health (4, 5). There is a continuous flow of hospitalizations and deaths attributed to extreme ambient temperatures (3, 6). Elevated short-term weather variations are known to be associated with morbidity and mortality, including heatwaves (3), cold exposure (7), and heavy rains and dust (8). Studies on the relationship between weather variations and mortality are common in high-income countries but limited in low- and middle-income countries.

Projections of future climate scenarios suggest that higher global mean temperatures could result in marked changes in the frequency of temperature extremes and could result in substantial increase in temperature-related mortality (9). Quantification of the population mortality burden attributable to adverse changes in weather conditions are essential for planning of adaptive approaches to minimize the impact of climate change on population, particularly in less developed countries in sub-Saharan Africa.

For instance, in Northern Ghana, drastic changes in weather conditions occur every year. The Harmattan season (December–February) is usually characterized by very cold weather conditions in the early morning followed by very hot temperature, sometimes above 40°C, during the day. On the contrary, during the peak of the wet season (July–August), the weather is usually characterized by heavy rainfall and flooding, sometimes destroying houses and displacing families. However, the extent of the association of these weather conditions with population health is currently unknown. Studies in Europe and North America have shown significant association between increases in mortality and elevated temperature, measured by maximum or minimum temperature, heat index, and sometimes, other weather indices (10). The elderly, young children, and the poor and the sick are particularly at risk (3, 11, 12).

Studies in tropical countries have also shown that there is strong seasonal variation in *Plasmodium falciparum* malaria infection incidence with the peak observed in August and September, which corresponds to the peak of the rainy season (13, 14). Therefore, a better understanding of the factors that increase mortality as a result of the association of temperature and precipitation are needed to adequately define high-risk groups among the population. In this study, we assessed the association between temperature and precipitation on all-cause mortality in Northern Ghana.

## Methods and materials

The study was conducted in the Kassena-Nankana District^1^ (KND) of the Upper East region of Ghana, which covers an area of 1,675 km^2^ in size, and is between latitude 10° 30′ and 11°00′ north and longitude 0°50′ and 1°30′ west of the equator. Ecologically, the area is in the guinea savanna belt, characterized by semi-arid conditions with the vegetation consisting of grassland interspersed with short trees. There are two main seasons, the dry and wet seasons, which are influenced by two main air masses – the North-East Trade winds (Harmattan air mass) and the South-Westerlies (Tropical Maritime air mass), respectively. During the dry season (November–April), these winds are usually dry and dusty as they originate from the Sahara Desert. Minimum and maximum temperature during the day can range from 38°C to 42°C and night temperatures can fall below 18°C. During such periods, precipitation is virtually absent due to low relative humidity, which rarely exceeds 20%. During the wet season (May–October), the district experiences the tropical maritime air mass, which comes along with precipitation. Annual precipitation figures are in the range of 850 mm and 950 mm.

Previous studies have described the health profile of the people in the district (15–18). Malaria and diarrhea continue to account for a large proportion of deaths (14, 19, 20), but a recent study has shown a gradual transition from infectious disease to non-communicable disease (15).

Mortality data for the study comes from the longitudinal population surveillance data of the Navrongo Health and Demographic Surveillance System (NHDSS). Starting with an initial census of the population in July 1993, the HDSS has continuously followed the population every 4 months, monitoring vital events, including births, deaths, and in and out migrations. Mortality variables were stratified by sex and four categorized age groups: (0–4), (5–19), (20–59), and (60+). Table 1 summarizes the mean, maximum, and minimum daily mortality in the dataset. Weather data (daily minimum, maximum temperature and daily precipitation data) from January 1995 to December 2010 were obtained from the Navrongo Meteorology Station. Daily mean temperature data were aggregated from daily minimum and maximum temperatures, an index designed to better estimate exposure as it uses multiple observations per day and so should be less prone to measurement error compared with other temperature indices (21).

**Table 1:** Summary statistics of daily mortality data by age and sex in the Kassena-Nankana Districts (KNDs) of Northern Ghana (1995–2010)

Population groups	Daily mortality count(*n*)	Mean deaths	SD	Min–max	Percent (%)
All	31,144	5.3	2.4	0.1–21.2	100
Age group					
0–4	8,551	1.5	1.3	0.0–11.0	27.5
5–19	2,315	0.4	0.5	0.0–4.6	7.4
20–59	9,417	1.6	1.1	0.0–7.8	30.2
60 +	10,861	1.9	1.2	0.0–9.1	34.9
					
Gender					
Female	14,812	2.5	1.5	0.0–14.0	47.6
Male	16,332	2.8	1.6	0.1–12.2	52.4

## Statistical analysis

We used a time series Poisson regression approach allowing for over-dispersion (for the mortality versus precipitation model) that included time-varying factors, such as time trend and season. We used a range of short-term lags of daily weather variables to assess the association between daily mean temperature and precipitation on mortality.

The daily mortality data from the NHDSS has some groupings of deaths on the 15th day of the month. This follows an earlier practice by field staff to record the 15th day of the month if the exact date of death could not be established. The date of death grouping was taken care of by replacing the deaths on the 15th day of each month with the monthly average. The remaining deaths for the given month were then distributed equally across the days of the month.

We fitted models to assess the association of mean daily temperature and precipitation on all-cause mortality by age groups and gender. We assumed that mortality patterns in the study area could be driven by a range of mediating factors (disease mechanisms) that are weather related. Adjustment for time trends and seasonality were done using natural cubic spline functions allowing for fixed (3) degrees of freedom (df) per year. The association of precipitation on daily mortality was statistically tested and found to be linear. We tested the sensitivity of the df of the smooth function of time trend by allowing for different df (i.e. 2, 3, 4, 6 and 8). Similarly, the df for the smooth function for the lag days temperature and precipitation variables were tested for sensitivity using df ranging from 2 to 6. We tested the association of 0–1, 2–6, 7–13, and 14–27 lag days of precipitation on mortality and chose to exclude results for 7–13 lag days for precipitation. Similarly, the lag day terms tested for association between temperature and mortality were of 0–1, 2–6, and 7–13. These lag strata are the mean of daily temperature and precipitation of lag day periods. Model fit was assessed using deviance statistic and Akaike Information Criterion (AIC) to arrive at a more parsimonious model. In addition, partial autocorrelation, heteroscedasticity and Q-Q plots were used to assess model assumptions.

We estimated the relative risks (RRs) for the association of daily mean temperature and precipitation with daily mortality using lag strata. The cumulative lag day's association for temperature was calculated for all lag days at the 25th and 75th percentile assuming linearity. The RRs for precipitation were fitted as linear terms, as suggested above. Days within the study period without data on temperature and precipitation were treated as missing values in the analysis. Data preparation and analysis were carried out using Stata version 11.2 (Stata Corp., College Station, TX), whereas regression models were fitted using the statistical software R version 2.12.1 Copyright (C) 2010 (The R Foundation for Statistical Computing).

## Results

**Fig. 1:**
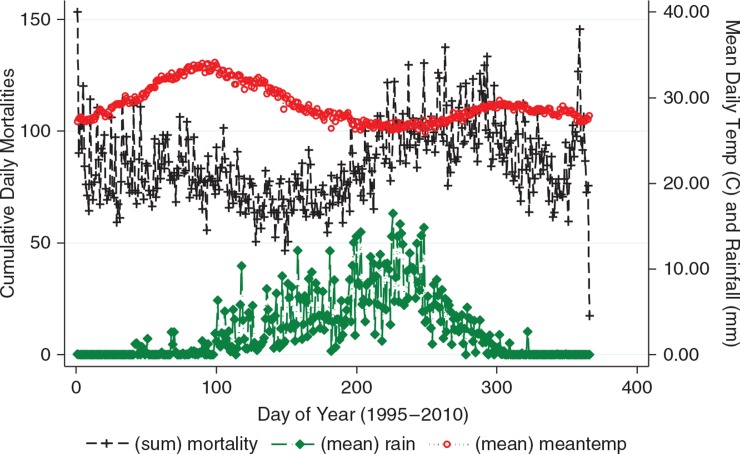
Seasonal pattern in daily mortality, daily mean temperature and precipitation in the Kassena-Nankana Districts (KNDs) of Northern Ghana (1995–2010).

The total deaths reported within the period (1995–2010) were 31,144. A longitudinal analysis shows that the study area has witnessed a general decline in all-cause mortality over the study period (results not shown). Most of the deaths occurred among the older population, that is, 60+ (34.9%), while the highest maximum daily deaths occurred among children younger than age 5(11.0). Average daily mortality was relatively raised round the 90th day of the year coinciding with the peak of the temperature period (March–April). However, the peak in mortality seems a little delayed compared with the peak in precipitation as shown in Fig. 1. Minimum and maximum daily temperature and precipitation are also shown in Table 2.

**Table 2:** Summary statistics of daily temperature and precipitation in the Kassena-Nankana Districts (KNDs) of Northern Ghana (1995–2010)

Variable	Mean	SD	Min	Max	25th	75th	% missing
Temperature (°C)							
Maximum	35.3	3.8	20.4	44.2	32.5	35.6	4.0
Minimum	23.0	2.8	12.1	33.0	21.3	23.0	4.0
Mean	29.2	2.7	21.4	37.0	27.3	30.8	4.0
Precipitation (mm)	3.0	9.3	0.0	93.0	0.0	0.0	5.5

### Association of daily mean temperature (°C) on daily mortality over lagged strata

**Fig. 2:**
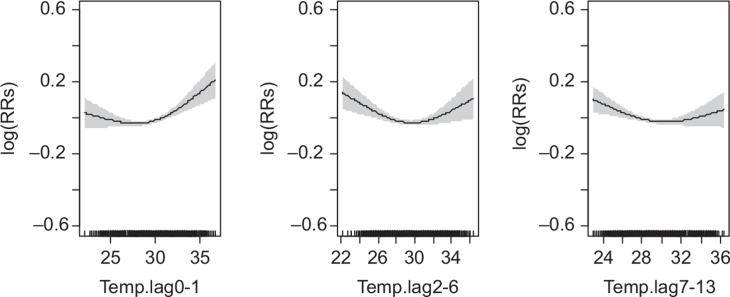
Relative risks (RRs) of daily mortality among age and sex groups with daily mean temperature over lagged strata. Gray regions are corresponding 95% confidence intervals.

The relationship between daily mean temperature and mortality is generally ‘U’ shaped for the short-term lag days of mean daily temperature (Fig. 2). There is a decrease in mortality from lower temperature up to about 30°C and then an increase upwards with increasing temperature. The thresholds for the 25th and 75th percentiles for the lag strata are 27.4°C and 30.6°C, respectively. The relationship tends to be linear for the association between temperature and mortality for lag days 2–6 among the 5–19 years age group and lag days 0–1 and 7–13 for the 20–59 years age group (results not shown).

For all populations, a statistically significant association of mean daily temperature on mortality at lag days 0–1 was observed below and above the 25th and 75th percentiles (*p*-value 0.029 and 0.008), respectively (Table 3). We also observed a statistically significant association of mean daily temperature above 75th (30.6°C) percentile for lag days 2–6 and 7–13 but not at the 25th percentile (27.4°C), as shown in Table 3. However, the cumulative effect of lag days at the 25th and 75th percentiles were 1.0% (95% confidence interval [CI]: −0.8%, 2.8%) and 1.8% (95% CI: 0.7%, 2.9%), respectively (Table 4).

**Table 3:** Percent increase (95% confidence interval [CI]) for all cause daily mortality associated with 1°C increase in mean daily temperature

Variable	Effect estimates			

	25th percentile		75th percentile	
		
Temperature (C)	% increase	95% CI	% increase	95% CI
All cause				
Lag 0–1	**0.19**	**(0.05, 0.21)**	**1.14**	**(0.12, 1.54)**
Lag 2–6	0.75	(−0.53, 1.94)	**0.32**	**(0.16, 0.25)**
Lag 7–13	0.06	(−1.21, 1.95)	**0.31**	**(0.14, 0.26)**
Males				
Lag 0–1	**0.22**	**(0.03, 0.28)**	1.11	(−0.23, 2.04)
Lag 2–6	0.82	(−0.86, 2.56)	**0.43**	**(0.21, 0.34)**
Lag 7–13	−0.04	(−1.72, 2.58)	**0.43**	**(0.21, 0.34)**
Female				
Lag 0–1	0.15	(−0.03, 0.28)	1.16	(−0.23, 2.11)
Lag 2–6	0.67	(−1.06, 2.64)	0.20	(−0.03, 0.35)
Lag 7–13	0.18	(−1.55, 2.66)	0.17	(−0.06, 0.35)
Age groups 0–4				
Lag 0–1	0.05	(−0.19, 0.36)	0.61	(−1.20, 2.78)
Lag 2–6	−0.13	(−2.37, 3.48)	**0.67**	**(0.36, 0.47)**
Lag 7–13	1.14	(−1.14, 3.51)	**0.50**	**(0.19, 0.47)**
Age groups 5–19				
Lag 0–1	0.34	(−0.05, 0.58)	2.35	(−0.40, 4.20)
Lag 2–6	3.08	(−0.40, 5.31)	0.38	(−0.05, 0.66)
Lag 7–13	0.63	(−2.77, 5.33)	**0.66**	**(0.22, 0.66)**
Age groups 20–59				
Lag 0–1	0.14	(−0.09, 0.35)	0.52	(−1.11, 2.50)
Lag 2–6	0.33	(−1.71, 3.15)	0.19	(−0.08, 0.40)
Lag 7–13	−0.39	(−2.42, 3.17)	0.14	(−0.13, 0.41)
Age groups 60 +				
Lag 0–1	**0.29**	**(0.07, 0.33)**	1.52	(−0.09, 2.45)
Lag 2–6	1.09	(−0.92, 3.08)	0.12	(−0.14, 0.40)
Lag 7–13	−0.63	(−2.62, 3.10)	0.19	(−0.07, 0.40)

Note: Bold figures show statistical significant results.

**Table 4:** Cumulative lag effect as a percent increase (95% confidence interval [CI]) for all cause mortality associated with 1°C increase in mean daily temperature

	Cumulative effect at 25th percentile for all lags		Cumulative effect at 75th percentile for all lags	
		
Category	% increase	95% CI	% increase	95% CI
All cause	1.00	(−0.82, 2.84)	**1.78**	**(0.73, 2.85)**
Male	0.99	(−1.39, 3.43)	**1.99**	**(0.60, 3.40)**
Female	1.00	(−1.45, 3.52)	**1.54**	**(0.11, 2.99)**
Age groups 0–4	1.06	(−2.15, 4.37)	1.79	(−0.09, 3.71)
Age groups 5–19	4.08	(−0.87, 9.27)	**3.42**	**(0.57, 6.35)**
Age roups 20–59	0.08	(−2.80, 3.05)	0.85	(−0.83, 2.56)
Age groups 60+	0.75	(−2.09, 3.68)	**1.84**	**(0.18, 3.53)**

Note: Bold figures show statistical significant results.

**Fig. 3:**
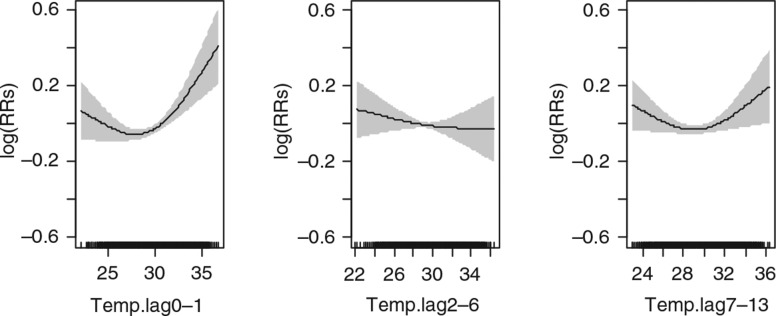
Relative risks (RRs) of daily mortality among children under 5 years of age with daily mean temperature over lagged strata. Gray regions are corresponding 95% confidence intervals.

The results also show that a 1°C increase in mean daily temperature above the 75th percentile at lag days 2–6 and 7–13 was significantly associated with an increase in risk of mortality among children younger than age 5 (Fig. 3), 0.7% (95% CI: 0.4%, 0.5%) and 0.5% (95% CI: 0.2%, 0.5%), respectively (Table 3).

Among children aged 5–19 years, temperature at the 75th percentile of lag days 7–13 was significantly associated with mean daily mortality 0.7% (95% CI: 0.2%, 0.7%).

**Fig. 4:**
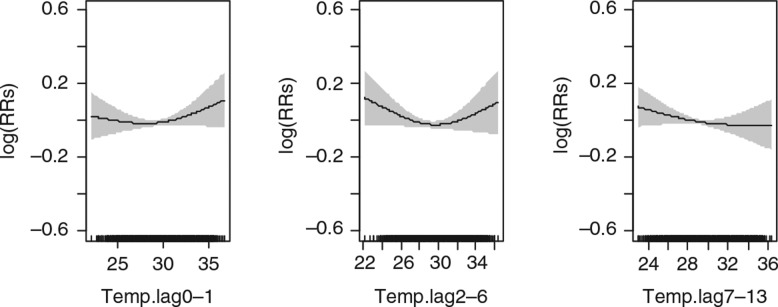
Relative risks (RRs) of daily mortality among adults 60 years and above with daily mean temperature over lagged strata. Gray regions are corresponding 95% confidence intervals.

Among the elderly adults, 60+, the only significant (10% level of significance) association of temperature was observed at lag days 0–1 below and above the 25th and 75th percentiles (Fig. 4). Among this age group, the cumulative lagged days’ associations were estimated to be 0.8% (95% CI: −2.1%, 3.7%) and 1.8% (95% CI: 0.2%, 3.5%), respectively, for the 25th and 75th percentiles (Table 3). Cumulatively, the lag days were not significantly associated with mortality below the 25th percentile 1.0% (95% CI: −1.5%, 3.5%) but significant at the 75th percentile 1.5% (95% CI: 0.1%, 3.0%) (Table 4).

Mortality among men seems to be higher per 1°C increase in daily mean temperature above the 30.6°C threshold for all the lag days but this was more pronounced in the 0–1 lag days (Table 3). Also, the cumulative association of the lag days strata were estimated to be 2.1% (95% CI: −1.4%, 3.4%) and 2.0% (95% CI: 0.6%, 3.4%), respectively, at the 25th and 75th percentiles (Table 4).

### Association of daily precipitation (mm) on daily mortality over lagged strata

**Fig. 5:**
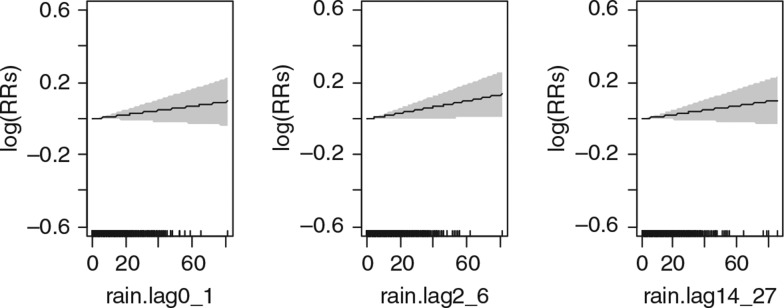
Relative risks (RRs) of daily mortality among all age and sex groups with daily precipitation over lagged strata. Gray regions are corresponding 95% confidence intervals.

Precipitation in the study area has a linear relationship with daily mortality (Fig. 5). The results show that short-term precipitation has an increasing association on mortality among the whole population (Table 5). A 10-mm increase in precipitation was found to be associated with a 1.7% (95% CI: 0.1%, 3.3%), at lag days 2–6 for the whole population (Table 5).

**Table 5:** Percent increase (95% confidence interval [CI]) for all cause daily mortality associated with 10 mm increased in precipitation

Variable	Linear effect	

Precipitation (mm)	%	95% CI
All cause		
Lag 0–1	1.19	(−0.46, 2.87)
Lag 2–6	1.71	(0.10, 3.34)
Lag 14–27	1.22	(−0.35, 2.81)
Males		
Lag 0–1	1.79	(−0.39, 4.00)
Lag 2–6	2.92	(0.80, 5.09)
Lag 14–27	2.35	(0.28, 4.45)
Female		
Lag 0–1	0.55	(−1.67, 2.82)
Lag 2–6	0.38	(−1.77, 2.59)
Lag 14–27	−0.02	(−2.13, 2.13)
Age groups 0–4		
Lag 0–1	0.42	(−2.39, 3.32)
Lag 2–6	1.36	(−1.34, 4.13)
Lag 14–27	1.84	(−0.75, 4.51)
Age groups 5–19		
Lag 0–1	−3.04	(−7.63, 1.78)
Lag 2–6	0.42	(−4.07, 5.12)
Lag 14–27	0.33	(−4.15, 5.03)
Age groups 20–59		
Lag 0–1	1.86	(−0.90, 4.70)
Lag 2–6	2.09	(−0.62, 4.88)
Lag 14–27	−0.24	(−2.90, 2.50)
Age groups 60 +		
Lag 0–1	2.11	(−0.43, 4.70)
Lag 2–6	1.92	(−0.57, 4.47)
Lag 14–27	1.78	(−0.64, 4.27)

For children younger than age 5, the association of a 10-mm increase in precipitation though was not observed to be significantly related with daily mortality, a higher risk was observed with increasing lagged days: 0.4% (95% CI: −2.4%, 3.3%), 1.4% (95% CI: −1.3%, 4.1%), and 1.8% (95% CI: −0.8%, 4.5%) for lag days 0–1, 2–6, and 14–27, respectively (Table 5).

Even though the association of 10 mm increase in precipitation at lag days 0-1, 2-6, and 14-27 strata among the age groups were found not to be statistically significant, the association of precipitation with gender presented mixed results. While mortality among females does not seem to be affected by both short-term and medium short-term lag days of precipitation, mortality among males seems to be more relatively affected by long-term lag days (between 2 and 4 weeks) (Table 5).

**Fig. 6:**
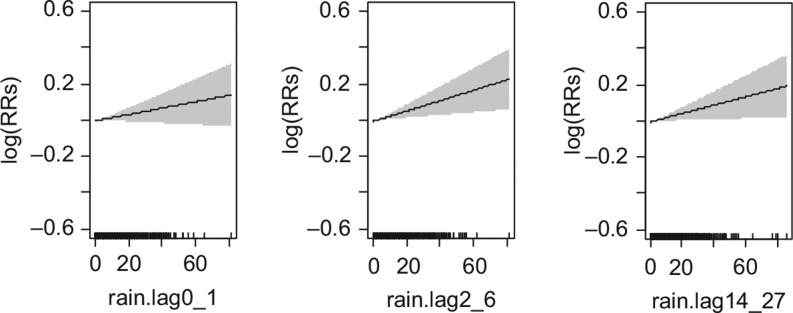
Relative risks (RRs) of daily mortality among males with daily precipitation over lagged strata. Gray regions are corresponding 95% confidence intervals.

For instance, daily mortality per a 10-mm increase in precipitation among females were observed to be 0.6% (95% CI: −1.7%, 2.8%), 0.4% (95% CI: −1.8%, 2.6%), and 0.0% (95% CI: −2.1%, 2.1%), respectively, at lags days 0–1, 2–6, and 14–27 (Table 5). Similarly for males, the association with mortality per a 10-mm increase in precipitation were 1.8% (95% CI: −0.4%, 4.0%) at lag days 0–1, 2.9% (95% CI: 0.8%, 5.1%) at lag days 2–6, and 2.3% (95% CI: 0.3%, 4.5%) at lag days 14–27 (Fig. 6 and Table 5).

## Discussion

Northern Ghana like other tropical climatic regions is characterized by seasonal variations in climatic conditions. However, there are no known studies or only few studies have examined the association between weather variation and mortality. The results show that both short-term daily mean temperature and long-term excess precipitation have a significant association on all-cause mortality in the whole population.

The cumulative association of temperature over the lag strata was estimated to be 1.8% higher per 1°C increase in daily mean temperature (above 30.6°C) on all-cause mortality across the whole population. Our study has corroborated well with previous studies showing that the elderly in society are among the most vulnerable groups during periods of increase in temperature (3, 11). For instance, Hales et al. (22) in their study found that a 1.8°F increase in temperature was associated with 1% (95% CI: 04%, 2.1%) in all-cause mortality.

Furthermore, we found that the association of temperature above the 75th percentile between lag weeks 1 and 2 was significant with a relative risk of 0.7% (95% CI: 0.2%, 0.7%) among children and young adolescents in the 5–19 age group. However, in the 20–59 age group, the effect was not significant.

Furthermore, we found that mortality related to long-term association of excess precipitation dramatically increased per a 10-mm increase in precipitation with an associated risk of 2.3% (95% CI: 0.3%, 4.5%) and 2.9% (95% CI: 0.8%, 5.1%) in the short-term and medium short-term lag days, respectively, among the male population. Higher precipitation patterns may increase the burden of mortality as a result of increase in the risk of infectious diseases, such as malaria, diarrhea, and respiratory infections. Coincidentally, incidences of these diseases among the vulnerable populations are heightened during the peak raining season. Malaria is the most widespread disease in the districts during this time of the year, affecting both infants and the elderly (14). In a previous study by Armah et al. (23), it was found that diarrhea and mortality also peak during the wet season and may explain the excess mortality that have been observed in our study. One previous study showed that any precipitation, 4 days prior, was significantly associated with an 11% increase in acute gastrointestinal illness visits to pediatric emergency department (24). In a study on the seasonal pattern of pneumonia-related mortality among children younger than age 5, Ye et al. (25) provided evidence that mortality in this age group in Nairobi's slums peaks during the rainy season. The explanations for this association are varied and as this study did not examine the associations between precipitation and cause-specific mortality, we are presently not in a position to explain the reasons for the association of precipitation with the male gender.

The study area has witnessed a couple of meningitis epidemics in recent years, mostly at a time when temperature is at its peak (March–April). Previous studies conducted in the study area have shown that more deaths attributable to meningitis have been observed in the dry seasons compared to any time of the year (26, 27). This could be explained by the long dry and hot season coupled with the housing architecture (poor ventilation) and sleeping arrangement of the population during these hot weather conditions. In a study by Scovronick et al. (28) in South Africa, the authors found that future mortality burdens would be reduced by over 50% (approximately 5,000 deaths annually) under a development policy that prioritizes the replacement of informal housing compared to one that prioritizes the replacement of traditional dwellings. Our results have highlighted the population groups that are most vulnerable to excess weather conditions, thus allowing for the formulation of better policy interventions to minimizing the adverse effects of climate change on population health in Northern Ghana.

There are some potential limitations to this study. First, some deaths in the HDSS data were recorded on the 15th day of the month when data collectors fail to establish the exact date of death. In the analysis, we addressed this problem by replacing the deaths on the 15th day of the month with the monthly average number of deaths and then redistributing the rest of the deaths equally across the days of the month. This methodological approach of handling age grouping has not been reported in the research literature and, therefore, needs further investigation and validation. Second, we also did not adjust for confounding factors, such as air pollution (visibility), on the association between temperature and mortality. Previous studies have been able to establish mediating effect of air pollution on the association between daily mean temperature and mortality (29, 30). Finally, the weather data contained about 4% missing values. However, these data were randomly distributed and adjusted for in the analysis and, therefore, could not affect the validity of our results.

## Conclusion

This study has explored the relationship between temperature and precipitation variability with all-cause mortality to inform climate adaptation interventions in Northern Ghana. We provide evidence of increase in mortality associated with short-term increase in daily mean temperature exposure, particularly during the dry season on vulnerable populations, especially the young and elderly. We also observed statistically significant increased mortality associated with excess precipitation at medium short-term among males. The RRs associated with increased short-term daily mean temperature above the upper threshold seems to be stronger than short-term cool association and thus poses a serious public health hazard in the light of the global warming.

These new results provide a good basis for further research to elucidate the health impact and risk factors related to weather and climate change in Northern Ghana. They also support the call to develop interventions to control human activities on climate change and establish stronger public health systems to handle the consequences that future adverse climatic conditions will have on the population's health in Northern Ghana.

## Footnotes

1 In 2008, a new district, Kassena-NankanaWest District, was carved out of the initial Kassena-Nankana District. Consequently, the NDSS now covers two districts.
